# Direct Suppression as a Mechanism for Controlling Unpleasant Memories in Daily Life

**DOI:** 10.1037/a0036518

**Published:** 2014-04-21

**Authors:** Charlotte S. Küpper, Roland G. Benoit, Tim Dalgleish, Michael C. Anderson

**Affiliations:** 1 MRC Cognition and Brain Sciences Unit, Cambridge, United Kingdom, and Department of Clinical Psychology and Psychotherapy, Freie Universität Berlin; 2 Department of Psychology, Harvard University; 3 MRC Cognition and Brain Sciences Unit, Cambridge, United Kingdom; 4 MRC Cognition and Brain Sciences Unit, Cambridge, United Kingdom, and Behavioural and Clinical Neurosciences Institute, University of Cambridge

**Keywords:** memory inhibition, intrusive memories, direct suppression, PTSD

## Abstract

Suppressing unwanted memories can impair their later recall. Recent work shows that this forgetting is achieved by at least two mechanisms supported by distinct neural systems: thought substitution and direct suppression (Benoit & Anderson, 2012). Here, we examined whether direct suppression, thought to be achieved by down-regulation of hippocampal activity, can disrupt memory of aversive scenes, and, if so, whether this disruption is linked to people’s perception of their ability to control intrusive thoughts. We presented participants with strong naturalistic reminders to aversive scenes and asked them to either covertly retrieve or directly suppress the associated scenes. Later, participants were cued with the reminders and asked to recall the scenes in detail. Direct suppression reduced recall probability of the scenes and also reduced the number of details recalled, even when scenes were remembered. Deficits in recall arose for minor details but also for details central to each scene’s gist. Participants with higher self-perceived control abilities over intrusive thoughts showed greater forgetting than did those reporting lower levels of control. These findings suggest that inhibitory processes underlying direct suppression can disrupt retention of aversive visual memories and link those processes to individual differences in control over intrusive thoughts in everyday life. These findings reinforce the possibility that inhibition may be less efficient in people likely to acquire posttraumatic stress disorder in the wake of a traumatic experience.

People sometimes encounter reminders of experiences that they would rather not think about. When this happens, they often try to stop retrieval to exclude the unwanted memory from awareness. The ability to suppress retrieval in this manner may play an important role in successfully adapting memory after a traumatic experience. Intrusive memories are common after traumatic life events (Brewin, Gregory, Lipton, & Burgess, 2010; Ehlers, Hackmann, & Michael, 2004), as are efforts to suppress them. Although intrusions gradually become less frequent for most people (Ehlers, 2010), for a significant minority, intrusions persist, causing significant distress and impairment (Ehlers et al., 2004; Kessler, Sonnega, Bromet, Hughes, & Nelson, 1995). Indeed, prolonged intrusions are the hallmark symptom of posttraumatic stress disorder (hereinafter, PTSD) and are considered a precursor to the disorder (American Psychiatric Association, 2000). A fundamental question is why many people learn to control intrusive memories over time, often recovering on their own, whereas others experience persistent intrusions that often require treatment. Studying the fundamental mechanisms of memory control may elucidate how people regulate intrusive memories and reveal the mechanisms of successful adaptation and of PTSD.

The ability to suppress retrieval of unwanted memories is often studied using the think/no-think (TNT) paradigm (Anderson & Green, 2001). In this task, participants first study cue–target pairs. They then either repeatedly retrieve (think items) or stop retrieval (no-think items) of the studied targets when given their reminder cues. A third group of items is neither retrieved nor suppressed (baseline items). After this think/no-think task, memory for all pairs is tested. A large body of data indicates that repeatedly stopping retrieval typically reduces recall for no-think items relative to think and baseline items on the final test (e.g., Anderson & Green, 2001; Anderson et al., 2004; Depue, Curran, & Banich, 2007; Levy & Anderson, 2012; for reviews, see Anderson & Hanslmayr, in press; Anderson & Huddleston, 2011). Although most previous studies with this procedure have used a general “no-think” instruction that does not specify how to stop retrieval, recent work has tried to isolate distinct forgetting mechanisms with more precise instructions. Benoit and Anderson (2012), for example, contrasted *thought substitution* (i.e., retrieving alternative associations to reminder cues) with *direct suppression* as ways of excluding a memory from awareness (Bergström, de Fockert, & Richardson-Klavehn, 2009). Direct suppression instructions specifically ask participants to not retrieve distracting thoughts and to purge memories from awareness if they intrude, effectively asking participants to shut down all retrieval in response to cues. Although both instructions led to similar forgetting, they engaged distinct neural systems: Whereas thought substitution engaged left ventrolateral prefrontal cortex to retrieve substitute memories, and its activation predicted increased retrieval-related activity in the hippocampus, effective connectivity analysis indicated that direct suppression recruited right dorsolateral prefrontal cortex to down-regulate hippocampal activity and inhibit conscious recollection (Benoit & Anderson, 2012). Thus, direct suppression instructions are thought to better isolate inhibitory control processes acting on hippocampally mediated traces than general no-think instructions, which may include some component of thought substitution.

Although it is possible that inhibitory control reduces traumatic intrusions (e.g., Aupperle, Melrose, Stein, & Paulus, 2012; Levy & Anderson, 2008; Wessel, Overwijk, Verwoerd, & de Vrieze, 2008), the case for whether the mechanisms engaged by direct suppression instructions impair complex, aversive memories is unclear. On the one hand, all direct suppression studies (Benoit & Anderson, 2012; Bergström et al., 2009; Hanslmayr, Leipold, Pastötter, & Bäuml, 2009) have used word pairs and only one (van Schie, Geraerts, & Anderson, 2013) has used emotional items. It is surely easier to suppress emotional words than traumatic intrusions. Traumatic intrusions often consist of brief images, rather than verbal thoughts (Ehlers et al., 2004; Holmes & Mathews, 2010), and recall of both aversive and pictorial stimuli is enhanced compared to that of neutral and verbal material (Holmes & Mathews, 2010; Nowicka, Marchewka, Jednorog, Tacikowski, & Brechmann, 2011; Payne & Corrigan, 2007; Quinlan, Taylor, & Fawcett, 2010; Shepard, 1967). On the other hand, no study showing suppression-induced forgetting for emotional material, such as autobiographical memories (Noreen & MacLeod, 2013), aversive scenes (Depue, Banich, & Curran, 2006; Depue et al., 2007), or negative word pairs (Hertel & McDaniel, 2010; Joormann, Hertel, Brozovich, & Gotlib, 2005; Joormann, Hertel, Lemoult, & Gotlib, 2009), has used direct suppression instructions, making it unclear how much forgetting was caused by thought substitution. More broadly, it remains unclear whether the inhibition process measured with direct suppression is engaged in naturally arising cases of memory control in everyday life.

To establish inhibitory control as a component process in how people contend with intrusive memories, it is important to show that, in principle, this process could be effective in controlling aversive memories in real life circumstances. The present study thus had two goals central to establishing direct suppression as a model of how people control unwanted memories in everyday life. First, we sought to determine whether direct suppression impairs retention of aversive visual memories that are strongly cued by reminders. In real traumatic situations, incidental objects present at the scene often become associated to the event, and when people encounter similar objects later, these can trigger unwanted memories (Ehlers & Clark, 2000; Ehlers et al., 2002). To model this feature, we modified Anderson and Green’s (2001) TNT paradigm and used materials adapted from those employed by Depue et al. (2007). Unlike Depue et al., however, we used object–scene rather than face–scene pairs, to increase the ecological validity of the suppression task. In particular, to simulate natural situations associated with involuntary trauma recall, we selected objects as cues that strongly resembled an incidental object embedded in its paired scene. Thus, cues were considerably more powerful and naturalistic than arbitrary pairs often used in TNT studies (see, however, Hertel & Gerstle, 2003; Hertel & McDaniel, 2010, for verbal studies that also create powerful cues via mental imagery encoding of emotional items).

Second, we examined the relationship between direct suppression and memory control abilities that people report experiencing in everyday life. Large variability exists in individuals’ perceptions about their ability to control unwanted intrusions (Luciano, Algarabel, Tomas, & Martinez, 2005), and self-report measures of this variation strongly predict symptoms such as anxiety, depression, and obsessional thinking (Peterson, Klein, Donnelly, & Renk, 2009; Williams et al., 2010). If these self-reports also predicted the forgetting induced by direct suppression in the TNT procedure, it would suggest both that direct suppression contributes to everyday memory control and that variations in it may be related to psychological symptoms. Such variation in suppression ability might be observed not merely in recall probability but also in the quality and specificity of memories (as shown, for example, by Fawcett, Taylor, & Nadel, 2013; Noreen & MacLeod, 2013; Stephens, Braid, & Hertel, 2012). Thus, we asked participants to describe their memories of the aversive scenes in detail on the final test. These descriptions allowed us to determine whether participants could recall the suppressed memories at all and whether suppression may affect the quality of the memories recalled in a graded fashion.

## Method

### Participants

Twenty-four native English speaking volunteers from the MRC Cognition and Brain Sciences Unit participant panel (age 18–30 years; *M* = 22.25 years; 18 female) participated in exchange for payment.

### Materials

**Figure 1 fig1:**
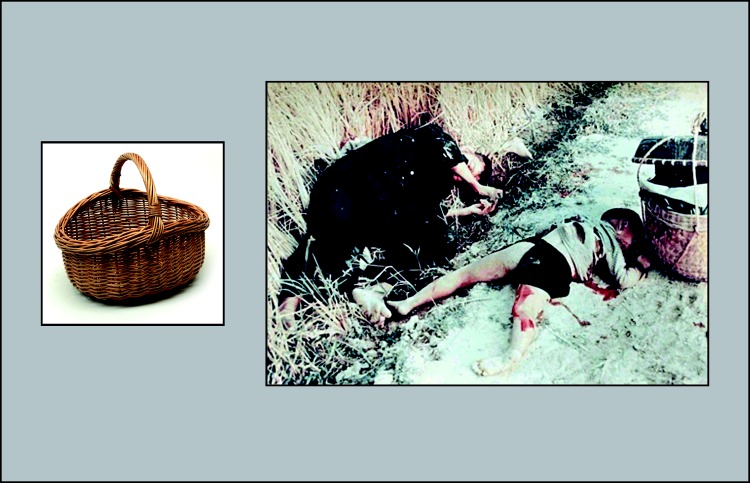
A representative cue–target pair, consisting of a negative scene and a neutral object that resembled an item embedded as a detail in its paired scene. Images are from the International Affective Picture System (Lang et al., 2008) and from Brady et al. (2008). See the online article for the color version of this figure.

The stimuli were 60 object–scene pairs. Scenes fit trauma themes such as combat exposure, physical and sexual assault, witnessing injuries and death, natural disasters, and serious accidents (see American Psychiatric Association, 2000; Kessler et al., 1995) and were taken from the International Affective Picture System (IAPS; Lang, Bradley, & Cuthbert, 2008) and online sources. Cues were photographs of familiar objects (taken from Brady, Konkle, Alvarez, & Oliva, 2008) set against a white background. Each object resembled an item embedded in its paired scene but was not intrinsically related to the scene’s gist, so that one could not guess the scene given the cue without having initially seen them together (see Figure 1). Of the 60 pairs, 12 served as fillers, with the remaining 48 constituting critical pairs (16 think, 16 baseline, 16 no-think items). The three sets were matched on valence and arousal of the scenes. Assignment of sets to the conditions was counterbalanced across participants.[Fig-anchor fig1]

#### Thought Control Ability Questionnaire (TCAQ; Luciano et al., 2005)

The TCAQ assesses the perceived ability to exert control over intrusions. Participants indicate whether they agree with each of 25 statements (e.g., “I manage to have control over my thoughts even when under stress” and “I get rid of uncomfortable thoughts or images almost effortlessly”) on a 5-point scale from 1 (*completely disagree*) to 5 (*completely agree*), with higher scores reflecting better control ability. The measure has a satisfactory test–retest reliability (*r* = .88) and a high internal consistency (Cronbach‘s alpha = .92).

### Procedure

**Figure 2 fig2:**
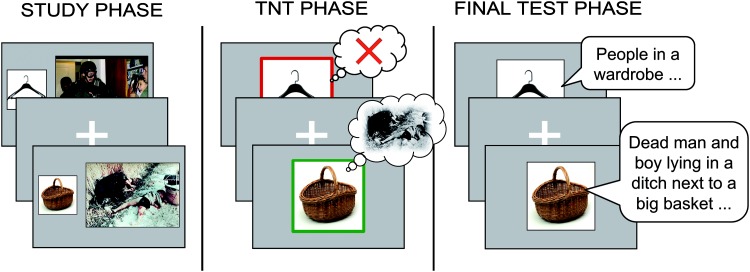
Schematic representation of the experimental procedure. In the study phase, participants encoded object–scene pairs. During the TNT phase, participants directly suppressed some of the scenes (no-think condition: top image; framed in red online only) and recalled others (think condition: bottom image; framed in green online only). In a final test, participants were asked to verbally describe all the scenes they had previously recalled, suppressed, or initially learned but not seen during the TNT phase (i.e., baseline items). TNT = think/no-think. Images are from the International Affective Picture System (Lang et al., 2008) and from Brady et al. (2008). See the online article for the color version of this figure.

The modified TNT procedure (Anderson & Green, 2001) consisted of three phases: a study phase, the TNT phase, and a final test phase (see Figure 2).[Fig-anchor fig2]

#### Study phase

Participants studied each object–scene pair for 6 s (interstimulus interval [ISI] = 1 s). Test–feedback cycles followed, in which each object appeared for up to 4 s (ISI = 750 ms) and participants indicated, by pressing a button, whether they could recall the scene. If they could, three scenes appeared, and they had to select the correct one within 4 s. Foil scenes were drawn from other studied pairs to ensure that a scene’s familiarity could not be used as a recognition cue. Participants then received the pair again for 2.5 s, as feedback. This procedure was repeated until participants reached 60% or higher recognition. To facilitate learning, the pairs were trained in two blocks of 30. After all pairs were trained, a final test was given without feedback, to assess which pairs had been learned.

#### Think/no-think phase

Participants next performed a TNT task composed of think and no-think trials. On all trials, a cue object appeared for 3 s, surrounded by a colored frame, followed by a varying fixation cross (ISI = 2 s ± 600 ms). For green-framed objects (think), participants were asked to retrieve the associated scene in as much detail as possible; for red-framed objects (no-think), they suppressed retrieval of the scene. On no-think trials, participants received direct suppression instructions (Benoit & Anderson, 2012; Bergström et al., 2009). Specifically, they were told to focus on the cue while blocking out all thoughts of the associated scene without engaging in distracting activity. Think and no-think cues were pseudo-randomly intermixed with the restriction that no four objects belonging to the same condition appeared in succession.

To assess understanding of the instructions, we asked participants to complete two short practice phases on fillers. They were then briefly refreshed (1.5 s exposure per pair) on all pairs before the TNT phase. During the TNT phase, participants were presented with 32 object cues, 16 each from the think and no-think conditions. The TNT phase was split into five blocks (separated by 30 s breaks) with each object presented twice in each block, yielding 10 think or no-think repetitions per object.

#### Final test phase

Finally, participants’ memory for all scenes was tested. Participants were first presented with six filler objects, followed by 48 critical cues (without a colored frame), with cues presented in a blocked randomization scheme. Each object was displayed for 15 s (ISI = 3 s). Participants were asked to recall every scene in as much detail as possible, describing each scene so that it could be uniquely identified. The descriptions were recorded.

After the test, participants rated, on a 5-point Likert scale (*never* = 0; *always* = 4), how often, overall, they used thought substitution and direct suppression for controlling their memories, and completed the TCAQ.

### Dependent Measures

Participants’ descriptions were scored on three measures. In the *identification* measure, a description was scored as correct if it included enough detail so that the scene could be identified (based on the measure introduced by Depue et al., 2006). In the *detail* measure, descriptions were divided into small segments that independently conveyed information, and the absolute number of correct details was counted. Finally, in the *gist* measure, we defined gist as any element pertaining to the scene’s story that could not be changed or excluded without changing the main theme. For each scene, two independent judges determined specific elements necessary to the gist (ranging from two to four per scene, *M* = 3.10, *SD* = 0.69), and a description was scored as correct only if it included all necessary elements. The descriptions were scored by two independent coders, both blind to the conditions. Interrater agreement, examined for a subsample of 11 participants, was high (identification: *r* = 1; detail: *r* = .91; gist: *r* = .98).

## Results

Only pairs that participants learned were analyzed (all relevant results were significant based on all pairs as well). Each measure was submitted to a repeated-measures analysis of variance (ANOVA) with condition (baseline, think, no-think) as a within-subject factor. Greenhouse–Geisser corrections were used where sphericity assumptions were violated as indicated by Mauchly‘s test. Significance level was set at α = .05.

### Final Test Performance

**Figure 3 fig3:**
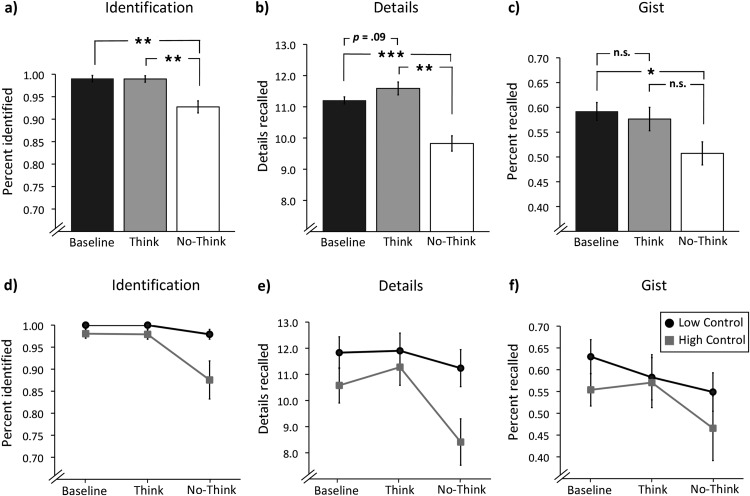
Memory performance in the final test as a function of baseline, think, and no-think conditions. (a) Percentage of memories correctly identified, (b) absolute number of details correctly recalled, and (c) percentage of correct gist recall for *n* = 24 participants. Graphs d–f show memory performance separately for participants with lower (*n* = 12) and higher (*n* = 12) control over everyday intrusions for the (d) identification, (e) details, and (f) gist measures. Error bars indicate ±1 standard error of the mean. Significance levels are represented as follows: *p < .05. **p < .01. ***p < .001. n.s. = not significant.

All three measures showed evidence of suppression-induced forgetting. On the identification measure, performance was extremely high, indicating that our object cues powerfully reinstated the scenes (see Figure 3a). Nevertheless, in line with previous findings (Depue et al., 2006, 2007), suppression reduced recall for no-think images (*M* = 93%) relative to baseline and think images (*M* = 99%, in each case). The ANOVA revealed a main effect of condition, *F*(1.08, 24.76) = 9.32, *p* = .005, η^2^ = .29, and a planned contrast showed that this partly reflected below baseline forgetting of no-think items, *F*(1, 23) = 9.72, *p* = .005, η^2^ = .30.[Fig-anchor fig3]

Importantly, participants also recalled fewer scene details (see Figure 3b), as revealed by a reliable main effect of condition, *F*(1.33, 30.50) = 14.92, *p* = .0001, η^2^ = .39 (*M* = 11.12, 11.51, and 9.76 for baseline, think, and no-think, respectively), and a planned contrast showing that participants recalled fewer details for no-think than baseline items, *F*(1, 23) = 18.25, *p* = .0001, η^2^ = .44. Strikingly, 88% of the participants showed this effect. Below baseline recall of details even arose when we considered only pairs that participants had correctly identified, *F*(1, 23) = 8.44, *p* = .008, η^2^ = .27, indicating that even accessible memories were recalled in less detail than they otherwise would have been.

Does suppression affect retention of a scene’s core meaning or only minor details? In our gist measure, we observed a trend for a main effect, *F*(2, 46) = 2.81, *p* = .07 (*M* = 59%, 58%, and 50% for baseline, think, and no-think, respectively), and, critically, no-think recall was reduced compared to baseline recall, *F*(1, 23) = 6.23, *p* = .020, η^2^ = .21. This effect was marginally significant when considering only correctly identified scenes, *F*(1, 23) = 3.67, *p* = .068, η^2^ = .14. Thus, suppression impaired retention of meaningful elements of the scenes (see Figure 3c).

### Variation by Reported Memory Control Ability

To determine whether forgetting is related to everyday perceptions of memory control, we split our sample into groups with higher (*M* = 96, *SD* = 9; *n* = 12) and lower (*M* = 66, *SD* = 16; *n* = 12) self-rated mental control abilities according to the Thought Control Ability Questionnaire. On the identification measure (see Figure 3d), high-control participants showed more below baseline forgetting (10.5%; *p* < .05) than did low-control participants (2.1%; *p* > .05), *F*(1, 22) = 5.13, *p* = .034, η^2^ = .13. Similarly, high-control participants showed more suppression of event details (2.2 details; *p* = .001) than did lower control participants (0.6 details; *p* = .05; see Figure 3e), *F*(1, 22) = 7.68, *p* = .011, η^2^ = .14. The groups did not differ in their suppression of gist (see Figure 3f). These results are echoed in the expected negative correlations of self-rated mental control with no-think recall for identification and for details (*r* = −.38 in both cases, *p* < .05, one-tailed) but not gist (*r* = −.1).^1^ Importantly, participants with low and high thought control ability did not differ on any measure of initial learning in the training phase (e.g., number of trials to learn pairs; recognition accuracy; judgments of recall; *p* > .7 in all cases). This indicates that differences were limited to memory control and not memory ability more generally.^2^

### Strategies for Memory Control

Participants reported following our instructions to use direct suppression. Participants were more likely to report using direct suppression (*M* = 3.04, *SD* = 0.55) than thought substitution (*M* = 1.15, *SD* = 0.77) during no-think trials, *t*(23) = 8.91, *p* = .000, *d* = 2.89.

## Discussion

The current study examined whether aversive images could be forgotten with direct suppression and, if so, whether such forgetting is related to people’s perceptions of their memory control skills in daily life. Recent work using effective connectivity analysis has isolated a right dorsolateral–hippocampal network supporting the direct suppression of episodic traces via inhibitory modulation of hippocampal retrieval processes (Benoit & Anderson, 2012; see also Gagnepain, Henson, & Anderson, 2014), supporting earlier claims about the mechanistic origins of reduced hippocampal activity during no-think instructions (Anderson et al., 2004; Depue et al., 2007). Although this mechanism likely contributed to prior instances in which emotional memory was suppressed (e.g., Chen et al., 2012; Depue et al., 2007), those studies used general no-think instructions (Depue et al., 2006, 2007; Hertel & McDaniel, 2010; Joormann et al., 2005, 2009; Noreen & MacLeod, 2013), making it unclear to what extent direct suppression mediated forgetting. Further, all studies using direct suppression instructions have used verbal materials (e.g., Benoit & Anderson, 2012; Bergström et al., 2009; van Schie et al., 2013), making it unclear whether aversive visual memories could be disrupted via this mechanism. By using direct suppression instructions and controlling thought substitution, and by using ecologically valid, potent reminders to aversive imagery, the present findings reinforce this earlier work and suggest the viability of inhibitory control as a mechanism for controlling intrusive imagery.

Prior neuroimaging studies with the think/no-think procedure have shown that direct suppression significantly reduces both hemodynamic and electrophysiological markers of episodic recollection. In contrast, retrieving thought substitutes does not cause such reductions (Benoit & Anderson, 2012) and in fact is associated with increases on these measures (Bergström et al., 2009). These findings indicate that, on average, participants do not use thought substitution when implementing direct suppression instructions. This conclusion is corroborated by participants’ post-experimental reports, which usually confirm very high compliance with the instruction to not retrieve thought substitutes during direct suppression. We also observed reports of good compliance in the present study. Nevertheless, because compliance is rarely perfect, and because the present study does not have neural indices that might detect retrieval of thought substitutes, it remains possible that some of the current suppression effect may reflect uncontrolled thought substitution. Given the similarity to prior procedures and findings, however, we suspect that thought substitution is unlikely to be a major factor, a possibility that will need to be corroborated with further imaging work.

Our findings further suggest that the inhibitory influence of direct suppression on hippocampal activity can also partially disrupt a memory, impairing its completeness and clarity, even when access to an event remains intact. Even when participants could generally recall the suppressed memory, they recalled fewer event details (cf. Fawcett et al., 2013; Noreen & MacLeod, 2013; Stephens et al., 2012). Degraded recall was not limited to minor details but also affected details central to the gist of the scenes. Such qualitative changes in memory may have clinical implications. For instance, one might speculate that the capacity to gradually weaken an unpleasant memory and to lose access to painful details by inhibitory control may gradually reduce distress, anxiety, and perceptions of uncontrollability among trauma survivors. Whether impaired recall of trauma is a benefit or a weakness is a matter of perspective, however. It may be important for trauma survivors to be able to choose to remember a traumatic experience (see Holmes, James, Kilford, & Deeprose, 2010). However, there are many situations in which it is unwise to discourage the use of suppression in regulating intrusions. For example, healthy people frequently exposed to traumatic situations (e.g., emergency service workers, military personnel), which they do not wish to intrude into their personal lives, would benefit from reducing recall of unpleasant experiences (Dunn, Billotti, Murphy, & Dalgleish, 2009).

Finally, the present findings link direct suppression to people’s naturally occurring efforts to control unwanted memories, and further suggest that there is wide variation in this ability that can be measured. By using a self-report measure of memory control ability known to predict individual differences in anxiety, depression, and obsessional thinking (Peterson et al., 2009; Williams et al., 2010), we found that participants with higher reported control over everyday intrusions were significantly more effective in suppressing aversive images than were participants with lower reported control. Such variation in ability may reflect underlying differences in the efficiency of the neural systems that implement direct suppression. Such variation might arise from differences in experience at memory control. Indeed, Lyoo et al. (2011) found that trauma survivors who showed the greatest cortical thickness in dorsolateral prefrontal cortex one year after the trauma also showed the largest reductions in PTSD symptom severity and significantly better recovery over several years. This raises the possibility that the frontohippocampal modulatory process exhibits experience-dependent plasticity, contributing to natural variation in memory control ability of the sort reported by our participants. Alternatively, such variation may reflect enduring differences in inhibitory control that portend the likely success a person will have in suppressing traumatic intrusions. Measuring and recognizing deficient memory suppression as a vulnerability factor may help to identify people who are not likely to recover on their own after a trauma and to guide appropriate intervention approaches (e.g., suppression- vs. acceptance-based) to prevent the buildup of the syndrome (Dunn et al., 2009; Peterson et al., 2009). Regardless of the source of individual variation, however, the link between naturally occurring memory control and the forgetting observed here suggests that understanding the cognitive and neural mechanisms underlying direct suppression offers exciting possibilities for future research on interventions to assist people recovering from traumatic life experiences.

## Footnotes

1 The correlation between the identification measure and self-reported control ability reported here reflects the exclusion of a single participant, whose no-think performance was 3.6 standard deviations below the mean. Excluding this participant does not affect the significance of any other relevant result.

2 Although the differences in memory suppression for participants with low and high thought control ability were reliable, the present study only had 24 participants, with 12 participants per group. As such, appropriate caution should be exercised in interpreting these findings, given the small sample size. However, in an independent, forthcoming study, we replicated the current differences in memory suppression as a function of self-perceived thought control ability. Indeed, when those participants are combined with the current ones (*N* = 42), all of the present conclusions are strongly supported.
